# Efficacy of a Topical Lanolin–Beeswax–Olive Oil Blend (RepoGen Cream) in Preventing Nipple Fissures in Breastfeeding Women: A Randomized Controlled Trial

**DOI:** 10.1111/jocd.70409

**Published:** 2025-08-28

**Authors:** Seyde Zeinab Modarresy, Reza Sattarpour, Parvaneh Sadeghimoghadam, Fatemeh Mollarahimi Maleki, Susan Belar, Azam Mohsennejad Saniani, Batool Sadat Hashemi, Maryam Noori

**Affiliations:** ^1^ Clinical Research of Development Unit, Hazrat‐e Fateme Masoume Hospital Qom University of Medical Sciences Qom Iran; ^2^ BreastFeeding Research Center, Family Health Institute, Imam Khomeini Hospital Complex Tehran University of Medical Sciences Tehran Iran; ^3^ Network of Immunity in Infection, Malignancy and Autoimmunity (NIIMA), Universal Scientific Education and Research Network (USERN) Tehran Iran; ^4^ Center for Health Related Social and Behavioral Sciences Research Shahroud University of Medical Sciences Shahroud Iran

**Keywords:** beeswax, breastfeeding, lanolin, nipple fissures, olive oil, randomized controlled trial, wound healing

## Abstract

**Background and Objectives:**

Pharmacological and non‐pharmacological treatments for nipple fissures have been investigated, with a variety of options yielding promising outcomes. Herbal treatments have also shown effectiveness, albeit with contradictory outcomes. Our objective was to evaluate the effectiveness of a topical blend containing lanolin, beeswax, and olive oil (RepoGen cream) in nipple fissure relief.

**Material and Methods:**

This study was a randomized, double‐blind, placebo‐controlled clinical experiment conducted at a teaching hospital in Qom, Iran, between March 2023 and 2024. Participants were mothers with singleton pregnancies who intended to breastfeed their newborns. The intervention group applied RepoGen cream to the nipple area before and after breastfeeding, while the control group applied placebo cream with no active ingredients. At 3 and 7 days postpartum, women and newborns were evaluated for nipple complications and breastfeeding behaviors and function.

**Results:**

A total of 133 mother‐infant dyads were enrolled, with 63 assigned to the RepoGen group and 70 to the control group. The RepoGen group demonstrated significantly lower nipple pain on both Day 3 (63.5% vs. 42.9%, *p* < 0.001) and Day 7 (84.1% vs. 71.4%, *p* = 0.01). The incidence of nipple wounds was also significantly reduced in the intervention group at both time points. Multivariate logistic regression analysis confirmed significantly lower odds of nipple pain, redness, wounds, and bleeding in the RepoGen group on Day 3, with sustained reductions in redness and wounds on Day 7. Although feeding frequency did not differ significantly between groups, the RepoGen group exhibited a significantly lower number of wet diapers on both days (*p* < 0.001). No maternal or neonatal adverse effects were reported throughout the study period.

**Conclusion:**

The current study confirmed the effectiveness of RepoGen cream for routine care in relieving nipple fissure symptoms among breastfeeding mothers without causing adverse effects. These findings suggest that RepoGen is a safe and effective intervention for improving maternal breastfeeding comfort during the early postpartum period.

## 1 Introduction

For breastfeeding mothers, breast fissures are a common and upsetting issue [1, 2]. Almost 80% of breastfeeding mothers develop nipple fissures. These conditions may lead to sores, swelling, blisters, redness, thinning of the skin, and changes in the nipple contour. If left untreated, nipple fissures can cause extreme pain, nipple bleeding, mastitis, and breast abscesses. They can also cause hematemesis and poor breastfeeding among newborns. Cracks often lead to early discontinuation of breastfeeding and impair mother's health [3, 4].

Lanolin, a natural lipid extracted from sheep's wool, is frequently utilized as a topical treatment for nipple discomfort and fissures. Lanolin comprises various lipids, including wax esters, sterols, and free fatty acids [5]. The emollient nature of this agent creates a protective layer on the skin, preventing the loss of moisture and enhancing the healing capabilities. Lanolin has been compared to treatments like *aloe vera*, hydrogel dressings, and expressed breast milk in multiple randomized controlled trials [5, 6], with some studies suggesting that lanolin provides no superior outcome on pain relief and healing time compared to other treatments [7, 8]. For instance, a study found olive oil may be more effective than lanolin in preventing irritation, while another found no difference in pain relief between lanolin and regular care, including routine hygiene and warmwater compresses, as per hospital protocol [8]. Like lanolin, beeswax has been used for topical wound healing for centuries [9]. It forms a semipermeable protective barrier that maintains a moist, beneficial environment for healing [10]. Additionally, beeswax contains vitamin A, which could promote epithelial formation and skin regeneration [11]. Thus, the lack of well‐designed standard trials makes clinicians hesitant to prescribe these medications.

RepoGen cream contains lanolin, beeswax, and olive oil, which may help to reduce nipple fissure symptoms and improve breastfeeding. Thus, the objective of our study was to evaluate the prophylactic effects of RepoGen in preventing nipple fissure symptoms, reducing nipple pain and redness, and improving breastfeeding outcomes among postpartum women.

## 2 Method

### 2.1 Study Design

This study was a randomized, double‐blind, placebo‐controlled clinical experiment conducted at the women's health and maternity center of Forghani Hospital in Qom, Iran, between March 2023 and March 2024.

### 2.2 Sample Size and Power Calculation

A priori sample size calculation was performed to ensure the study was sufficiently powered to detect a clinically significant difference. Based on previous studies [7, 8, 12], we anticipated a reduction in nipple fissure symptoms from approximately 40% in the control group to 20% in the RepoGen group. Using a two‐sided chi‐square test with *α* = 0.05 and power (1–*β*) = 0.80, we calculated that a minimum of 60 participants per group (total: *n* = 120) would be required to detect this difference. To account for an estimated 10% dropout or loss to follow‐up, we aimed to recruit at least 132 participants. Ultimately, 133 participants were enrolled and randomized, which met our target sample size and ensured adequate statistical power for the primary outcome.

### 2.3 Participants

The inclusion criteria targeted mothers admitted for delivery and who had singleton pregnancies. Only mothers who intended to breastfeed their newborns were considered for participation. Some exclusion criteria were set to ensure participant health and data authenticity. Mothers who were unwilling to take part and those who had breast deformities, such as flat or inverted nipples, or who had undergone breast‐shaping procedures were ruled out. Volunteers were excluded if they had a history of sensitivity or allergy to topical treatments, maternal diabetes, or other internal diseases, behavioral or psychological disorders, or if their neonates presented with orofacial anomalies. Finally, any other condition of the mother or the newborn that required cessation of breastfeeding led to exclusion from the study.

### 2.4 Randomization and Blinding

Everyone who took part received an identifying number. All data were collected and stored anonymously. To ensure randomization, the names of the two treatment groups, Group A and Group B, were randomly assigned by drawing from a pool. Once the groups were designated and the patient consented to participate in the study, each participant randomly chose one of the treatment groups by drawing the name of the treatment from a separate pool. This process was performed by an independent researcher who was not involved in the study procedures or data analysis. Both participants and investigators were blinded to the group assignments. Furthermore, participants in the control group were allowed to switch to RepoGen cream after 4 weeks if they found it effective and compelling, allowing them to benefit from the treatment while contributing to the research goals.

### 2.5 Intervention and Assessment Protocol

Following randomization, the intervention group applied RepoGen cream [13], an herbal topical formulation (Pars Azmaye Teb Company, Tehran, Iran; license no. 1584, batch no. 202301, manufactured March 2023, expiration March 2025) to the nipple area before the first breastfeeding and after each subsequent feeding. RepoGen nipple cream contains lanolin, beeswax, and olive oil and did not require removal prior to breastfeeding, due to its nontoxic and herbal composition. The product used in this study is commercially available under the name Genobiotic Nipple Crack Cream, and more information can be found on the manufacturer's official website: https://parsazmayeteb.com/product/genobiotic‐nipple‐crack‐cream/.

In comparison, the control group received a placebo cream identical in appearance, texture, and packaging to RepoGen cream but without active ingredients. Both participants and outcome assessors were blinded to group allocation. After 3 days, women and their newborns were evaluated in person by a trained observer blinded to group assignment and then by phone on Day 7 for nipple complications and breastfeeding behaviors and functions. Adherence to cream application was monitored via daily logs filled by researchers and also nursing notes.

### 2.6 Outcome Measures

The primary endpoint of this trial was the severity of nipple pain, assessed on Days 3 and 7 postpartum, using the Visual Analog Scale (VAS) ranging from “none” to “very severe” [14, 15]. Pain was selected as the primary outcome due to its clinical relevance as the most immediate and impactful symptom of nipple fissure during breastfeeding. Secondary endpoints included other local symptoms and breastfeeding‐related outcomes. These comprised the presence of nipple redness, wound, bleeding, and itching on Days 3 and 7. Additional secondary outcomes related to breastfeeding function included daily breastfeeding frequency, side preference, feeding sufficiency (assessed by the number of wet diapers and defecations per day), use of formula, and breast pump usage. These secondary outcomes were chosen to capture both clinical signs of healing and functional aspects of breastfeeding success.

### 2.7 Safety Profile and Adverse Effects

Safety was monitored throughout the study via direct observation on Day 3 and follow‐up interviews on Day 7. Mothers were instructed to report any signs of skin erythema, pruritus, edema, or signs of contact dermatitis and allergic reactions at the application site. Also, neonatal well‐being was tracked by recording any adverse events such as feeding intolerance, skin rashes, gastrointestinal disturbances, or changes in urine and stool output. Specific questions addressing maternal and neonatal adverse effects were included in the structured follow‐up.

### 2.8 Statistical Analysis

Demographic information was summarized using descriptive analysis, with frequencies and percentages reported for categorical variables. Group comparisons for categorical variables were conducted using the chi‐square test or Fisher's exact test, as appropriate. To evaluate the effect of the intervention while controlling for potential confounders, we applied logistic regression analyses. Binary logistic regression was used for dichotomous outcomes, ordinal logistic regression for ordered categorical outcomes, and multinomial logistic regression for outcomes with more than two unordered categories. All models were adjusted for maternal and neonatal demographic characteristics, including maternal age, body mass index (BMI), gravidity, gestational age, mode of delivery, infant gender, and infant birth weight. All analyses were performed using SPSS software (version 22, Chicago), and a two‐tailed *p*‐value < 0.05 was considered statistically significant.

### 2.9 Ethics

The Ethics Committee of Qom University of Medical Sciences approved this study (Ethics code: IR.MUQ.REC.1402.067, IRCT code: IRCT20230606058394N1). Before enrollment, all participants were required to give written informed consent, which was obtained after a comprehensive explanation of the study's protocols, interventions, objectives, and possible adverse effects. The study design was also based on the Helsinki declaration.

## 3 Results

**FIGURE 1:**
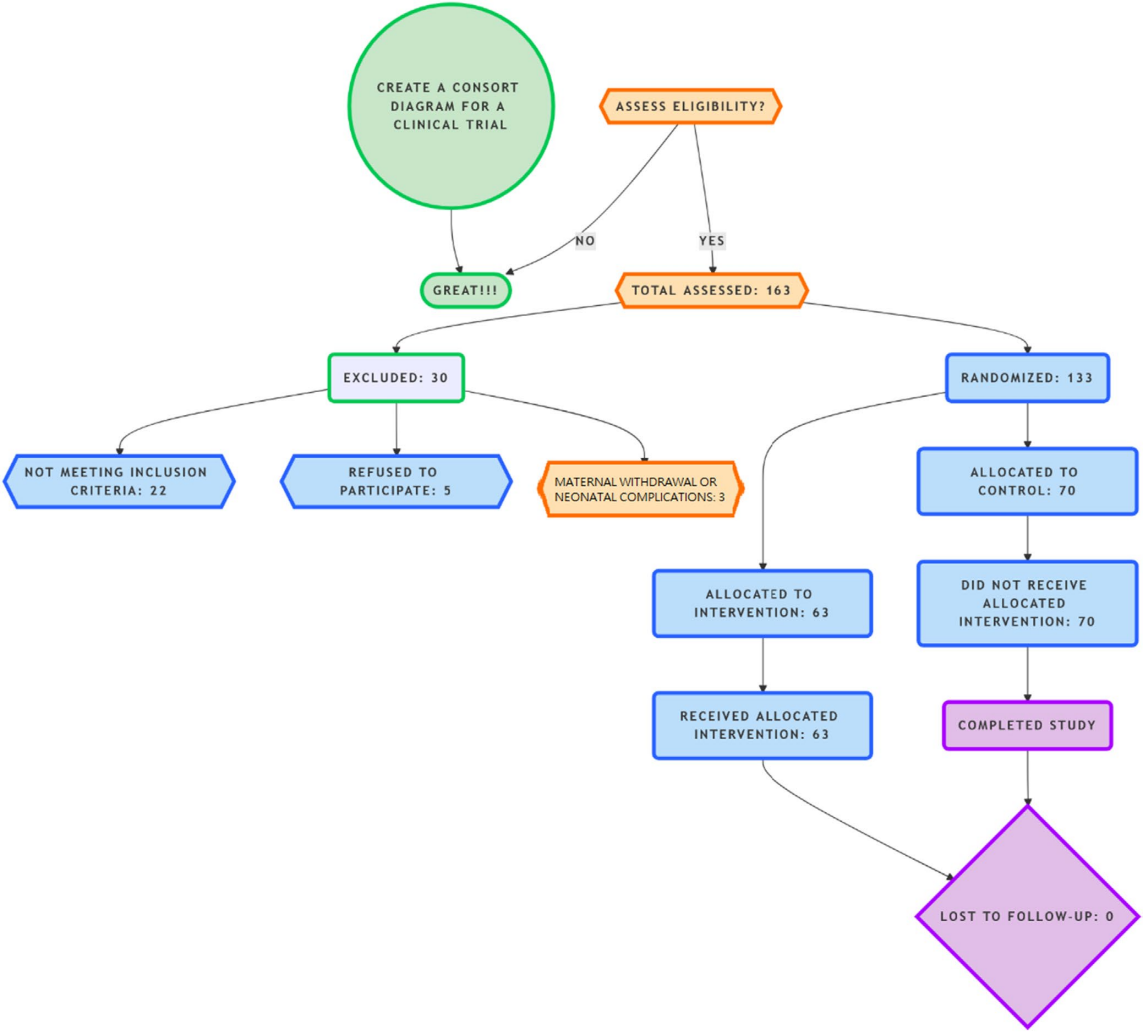
CONSORT diagram of the study flow.

One hundred sixty‐three participants were initially chosen for the study; 22 of them did not meet inclusion criteria, five refused to participate, and three of them were excluded because of maternal withdrawal or neonatal complications. Eventually, a total of 133 mother‐infant dyads were enrolled (Figure 1).

### 3.1 Baseline Characteristics

Table 1 demonstrates the descriptive characteristics of participants. Of the 133 participants surveyed, 63 were divided into the RepoGen group and 70 into the control group. Maternal age was similarly distributed, with overall 11.3% under 20 years, 42.9% aged 20–30 years, and 45.9% aged 30–40 years (*p* = 0.185). Also, maternal BMI showed a significant difference (*p* < 0.001); overweight status was more common in the RepoGen group (50.8%) compared to the control group (22.9%), while normal BMI was more frequent in the control group (75.7%). Vaginal delivery occurred in 64.7% overall, with a higher rate in the RepoGen group (76.2% vs. 54.3%; *p* = 0.008). Additionally, gravidity was significantly different (*p* = 0.028); primigravid mothers were more prevalent in the RepoGen group (38.1% vs. 15.7%). Lastly, infant gender distribution varied (*p* = 0.022), with more male infants in the RepoGen group (55.6% vs. 35.7%). Mothers' nationality (*p* = 0.283), gestational age at delivery (*p* = 0.786), or infant birth weight (*p* = 0.845) categories were balanced across groups (all *p* > 0.05).

**TABLE 1:** Baseline characteristics of included participants.

Characteristic	Category	Total, *n* (%)	Case (*n* = 63), *n* (%)	Control (*n* = 70) *n* (%)	*p*
Mother's age (years)	< 20	15 (11.3%)	7 (11.1%)	8 (11.4%)	0.185
	20–30	57 (42.9%)	32 (50.8%)	25 (35.7%)	
	30–40	61 (45.9%)	24 (38.1%)	37 (52.9%)	
Nationality	Iranian	84 (63.2%)	43 (68.3%)	41 (58.6%)	0.283
	Non‐Iranian	49 (36.8%)	20 (31.7%)	29 (41.4%)	
BMI	Underweight	4 (3.0%)	3 (4.8%)	1 (1.4%)	< 0.001
	Normal	81 (60.9%)	28 (44.4%)	53 (75.7%)	
	Overweight	48 (36.1%)	32 (50.8%)	16 (22.9%)	
Gestational age (weeks)	< 35	3 (2.3%)	2 (3.2%)	1 (1.4%)	0.786
	35–40	108 (81.2%)	51 (81.0%)	57 (81.4%)	
	> 40	22 (16.5%)	10 (15.9%)	12 (17.1%)	
Delivery type	NVD	86 (64.7%)	48 (76.2%)	38 (54.3%)	0.008
	C/S	47 (35.3%)	15 (23.8%)	32 (45.7%)	
Gravidity	1	35 (26.3%)	24 (38.1%)	11 (15.7%)	0.028
	2	44 (33.1%)	18 (28.6%)	26 (37.1%)	
	3	39 (29.3%)	14 (22.2%)	25 (35.7%)	
	> 3	15 (11.3%)	7 (11.1%)	8 (11.4%)	
Infant's gender	Male	60 (45.1%)	35 (55.6%)	25 (35.7%)	0.022
	Female	73 (54.9%)	28 (44.4%)	45 (64.3%)	
Infant's birth weight (g)	< 2500	11 (8.3%)	5 (7.9%)	6 (8.6%)	0.845
	2500–3000	25 (18.8%)	10 (15.9%)	15 (21.4%)	
	3000–3500	62 (46.6%)	30 (47.6%)	32 (45.7%)	
	> 3500	35 (26.4%)	18 (28.6%)	17 (24.3%)	

Abbreviations: BMI, body mass index; C/S, cesarean section; NVD, natural vaginal delivery.

### 3.2 Clinical Outcomes on Days 3 and 7

**FIGURE 2:**
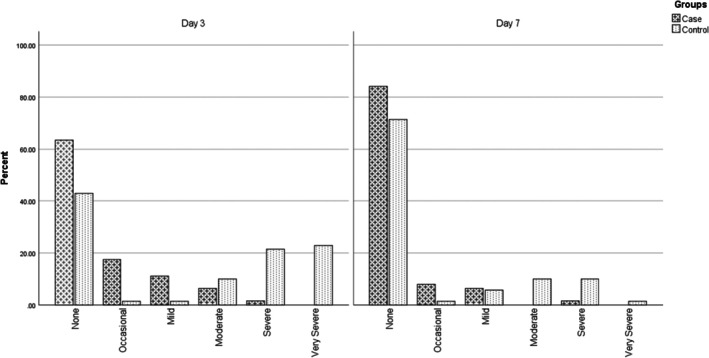
Nipple pain severity on Days 3 and 7 in the case and control groups. The chart shows pain severity distributions across both groups. On Day 3, the RepoGen group had significantly more pain‐free participants than the control group (63.5% vs. 42.9%, *p* < 0.001). By Day 7, the difference remained in favor of RepoGen (84.1% vs. 71.4%, *p* = 0.01), indicating reduced pain severity over time.

Nipple pain severity was significantly lower in the RepoGen group on both Days 3 and 7. On Day 3, 63.5% of mothers in the RepoGen group reported no pain compared to 42.9% in the control group (*p* < 0.001). This improved further by Day 7, with 84.1% in the intervention group being pain‐free compared to 71.4% in the control group (*p* = 0.01) (Figure 2).

**FIGURE 3:**
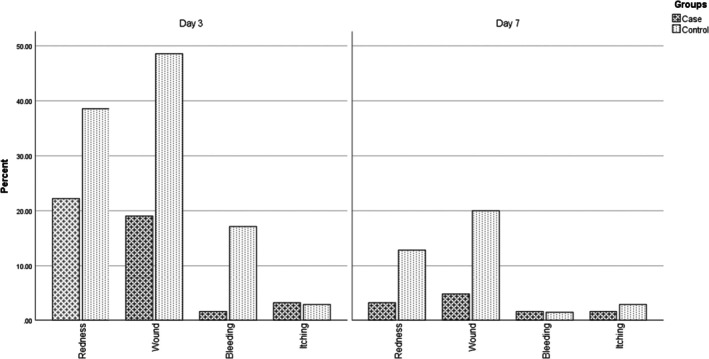
Nipple complications on Days 3 and 7 in the case and control groups. The chart compares the number of participants with redness, wound, bleeding, and itching across groups and time points. On Day 3, the RepoGen group showed significantly lower rates of wound (*p* < 0.001) and bleeding (*p* = 0.006), with a nonsignificant trend toward less redness (*p* = 0.064). On Day 7, wounds (*p* = 0.018) remained significantly less frequent in the RepoGen group. Itching rates were low and not significantly different.

Although not statistically significant, nipple redness was less frequent in the RepoGen group on both assessment days. On Day 3, 22.2% of participants in the intervention group had nipple redness versus 38.6% in the control group (*p* = 0.064). By Day 7, only 3.2% of the RepoGen group reported redness compared to 12.9% in the control group (*p* = 0.087) (Figure 3).

Nipple wounds were significantly more prevalent in the control group than the RepoGen group at both time points. On Day 3, 48.6% of the control group had nipple wounds, compared to 19.0% in the RepoGen group (*p* < 0.001). This difference persisted on Day 7 (20.0% vs. 4.8%, *p* = 0.018) (Figure 3).

Nipple bleeding was reported by only one mother (1.6%) in the RepoGen group on each day, whereas 12 mothers (17.1%) in the control group reported bleeding on Day 3 (*p* = 0.006). On Day 7, the rates were similar and very low in both groups (*p* = 1.0) (Figure 3).

Nipple itching was rare and not significantly different between groups on either day (Day 3: *p* = 1.000; Day 7: *p* = 1.000), with fewer than 3% of participants affected in either group (Figure 3).

Mothers in the RepoGen group were less likely to breastfeed more than 10 times daily on Day 3 compared to the control group (68.3% vs. 90.0%, *p* = 0.006); however, this difference was not statistically significant by Day 7 (85.7% vs. 95.7%, *p* = 0.116).

Use of formula feeding and breast pumps did not differ significantly between groups at either time point. Regarding breastfeeding side preference, all mothers in the control group used both breasts for feeding at both time points. In contrast, a notable proportion of the RepoGen group reported a preference for either the left or right breast: 38.1% on Day 3% and 19.0% on Day 7 (both *p*‐values < 0.001).

Based on bowel movement frequency, nearly all infants in both groups had adequate defecation. However, feeding sufficiency as measured by wet diaper count showed marked differences: 44.4% of infants in the RepoGen group had fewer than six wet diapers per day on Day 3, compared to none in the control group (*p* < 0.001). This difference persisted on Day 7 (30.2% vs. 0%, *p* < 0.001). Table 2 summarizes the outcome difference between the two groups.

**TABLE 2:** Comparison of clinical outcomes between case and control groups on Days 3 and 7 postpartum.

Variable	Category	Day 3			Day 7		
		Case, *n* (%)	Control, *n* (%)	*p*	Case, *n* (%)	Control, *n* (%)	*p*
Nipple pain	None	40 (63.5%)	30 (42.9%)	< 0.001	53 (84.1%)	50 (71.4%)	0.01
	Occasional	11 (17.5%)	1 (1.4%)		5 (7.9%)	1 (1.4%)	
	Mild	7 (11.1%)	1 (1.4%)		4 (6.3%)	4 (5.7%)	
	Moderate	4 (6.3%)	7 (10.0%)		0 (0.0%)	7 (10.0%)	
	Severe	1 (1.6%)	15 (21.4%)		1 (1.6%)	7 (10.0%)	
	Very severe	0 (0.0%)	16 (22.9%)		0 (0.0%)	1 (1.4%)	
Nipple redness	Yes	14 (22.2%)	27 (38.6%)	0.064	2 (3.2%)	9 (12.9%)	0.087
	No	49 (77.8%)	43 (61.4%)		61 (96.8%)	61 (87.1%)	
Nipple wound	Yes	12 (19.0%)	34 (48.6%)	< 0.001	3 (4.8%)	14 (20.0%)	0.018
	No	51 (81.0%)	36 (51.4%)		60 (95.2%)	56 (80.0%)	
Nipple bleeding	Yes	1 (1.6%)	12 (17.1%)	0.006	1 (1.6%)	1 (1.4%)	1
	No	62 (98.4%)	58 (82.9%)		62 (98.4%)	69 (98.6%)	
Nipple itching	Yes	2 (3.2%)	2 (2.9%)	1	1 (1.6%)	2 (2.9%)	1
	No	61 (96.8%)	68 (97.1%)		62 (98.4%)	68 (97.1%)	
Breastfeeding frequency (times daily)	< 5	2 (3.2%)	0 (0.0%)	0.006	1 (1.6%)	0 (0.0%)	0.116
	5–10	18 (28.6%)	7 (10.0%)		8 (12.7%)	3 (4.3%)	
	> 10	43 (68.3%)	63 (90.0%)		54 (85.7%)	67 (95.7%)	
Formula feeding	Yes	7 (11.1%)	10 (14.3%)	0.774	7 (11.1%)	10 (14.3%)	0.774
	No	56 (88.9%)	60 (85.7%)		56 (88.9%)	60 (85.7%)	
Breast pump using	Yes	4 (6.3%)	9 (12.9%)	0.332	6 (9.5%)	2 (2.9%)	0.149
	No	59 (93.7%)	61 (87.1%)		57 (90.5%)	68 (97.1%)	
Breastfeeding side preference	Both	39 (61.9%)	70 (100.0%)	< 0.001	51 (81.0%)	70 (100.0%)	< 0.001
	Left	11 (17.5%)	0 (0.0%)		9 (14.3%)	0 (0.0%)	
	Right	13 (20.6%)	0 (0.0%)		3 (4.8%)	0 (0.0%)	
Feeding sufficiency (Defecation/Day)	< 5	63 (100.0%)	70 (100.0%)	1	60 (95.2%)	70 (100.0%)	0.104
	5–10	0 (0.0%)	0 (0.0%)		3 (4.8%)	0 (0.0%)	
Feeding sufficiency (Wet diapers/Day)	< 6	28 (44.4%)	0 (0.0%)	< 0.001	19 (30.2%)	0 (0.0%)	< 0.001
	6–10	35 (55.6%)	70 (100.0%)		44 (69.8%)	70 (100.0%)	

Multivariate logistic regression was conducted to adjust for potential confounding variables. On Day 3, the odds of experiencing higher nipple pain in the RepoGen group were significantly lower (odds ratio [OR] = 0.132, 95% confidence interval [CI]: 0.054–0.319, *p* < 0.001). Similarly, the odds of nipple redness (OR = 0.151, 95% CI: 0.047–0.489, *p* = 0.002) and nipple wounds (OR = 0.140, 95% CI: 0.046–0.421, *p* < 0.001) were significantly reduced in the RepoGen group compared to the control group. The likelihood of nipple bleeding was also markedly lower in the RepoGen group on Day 3 (OR = 0.022, 95% CI: 0.001–0.484, *p* = 0.015). On Day 7, the RepoGen group continued to show significantly lower odds of nipple redness (OR = 0.130, 95% CI: 0.018–0.962, *p* = 0.046) and nipple wounds (OR = 0.211, 95% CI: 0.045–0.998, *p* = 0.050), while the reduction in nipple pain approached statistical significance (OR = 0.394, 95% CI: 0.142–1.095, *p* = 0.074). No significant differences were observed between groups regarding nipple bleeding on Day 7.

Feeding sufficiency, measured by the number of wet diapers per day, was significantly lower in the RepoGen group at both time points, with ORs of 0.019 (95% CI: 0.000–0.191, *p* < 0.001) on Day 3 and 0.038 (95% CI: 0.000–0.468, *p* = 0.006) on Day 7. No statistically significant associations were found between group allocation and other variables, including breastfeeding frequency, side preference, formula feeding, breast pump use, or defecation‐based feeding sufficiency (Table 3).

**TABLE 3:** Multivariate logistic regression analysis of clinical outcomes comparing case and control groups on Days 3 and 7.

Variable	Day 3		Day 7	
	OR (95% CI)	*p*	OR (95% CI)	*p*
Nipple pain	0.132 (0.054–0.319)	< 0.001	0.394 (0.142–1.095)	0.074
Nipple redness	0.151 (0.047–0.489)	0.002	0.130 (0.018–0.962)	0.046
Nipple wound	0.140 (0.046–0.421)	< 0.001	0.211 (0.045–0.998)	0.050
Bleeding	0.022 (0.001–0.484)	0.015	0.469 (0.010–21.490)	0.698
Itching	0.464 (0.012–17.344)	0.677	0 (0‐inf)	0.998
Breastfeeding frequency	0.864 (0.505–1.479)	0.594	0.954 (0.554–1.639)	0.864
Breastfeeding side preference				
Left vs. Both	1.052 (0.312–3.547)	0.934	1.051 (0.288–3.837)	0.940
Right vs. Both	1.074 (0.341–3.379)	0.904	1.076 (0.244–4.736)	0.923
Formula feeding	1.187 (0.335–4.202)	0.791	0.842 (0.231–3.060)	0.794
Breast pump using	0.607 (0.143–2.574)	0.499	2.470 (0.320–19.055)	0.386
Feeding sufficiency (Defecation/Day)	—	—	3.430 (0.199–194.889)	0.374
Feeding sufficiency (Wet diapers/Day)	0.019 (0.000–0.191)	< 0.001	0.038 (0.000–0.468)	0.006

### 3.3 Adverse Effects

No maternal or neonatal adverse effects were reported during the study period. None of the participants discontinued the intervention due to adverse reactions, and no skin irritation, allergic responses, or feeding complications attributable to the use of RepoGen cream were documented. No loss to follow‐up or missed application was noted.

## 4 Discussion

Our study found that topical application of RepoGen cream significantly reduces nipple pain, redness, wounds, and bleeding in postpartum mothers compared with placebo on the third day. On the seventh day, the superior effect of RepoGen cream in reducing redness and wounds was consistent, offering evidence that the cream is an effective and practical option for managing early postpartum nipple complications. Therefore, we suggest using a blend of lanolin, beeswax, and olive oil could potentially be beneficial for breastfeeding mothers facing nipple fissure symptoms.

Furthermore, feeding sufficiency, as measured by wet diaper frequency, was significantly lower in the RepoGen group on both days. While this finding warrants attention, it should be interpreted cautiously. The overall rates of formula use, pump usage, and infant defecation frequency were not significantly different between groups; no clinical signs of dehydration or poor feeding were reported. It is plausible that this result reflects reporting inconsistencies, overestimation by control mothers, or differential adherence to diaper tracking. Nonetheless, close monitoring of infant hydration status is recommended in future trials using RepoGen cream.

Our findings also support the safety profile of RepoGen cream, as no adverse effects were observed in either mothers or newborns, despite its repeated topical use and potential for incidental ingestion by infants during breastfeeding. Therefore, the cream had an excellent tolerability profile, with no safety issues requiring modification of the study protocol or additional medical management.

### 4.1 Pharmacological and Non‐Pharmacological Treatments

The focus of studies for nipple fissure treatment includes both pharmacological and non‐pharmacological options. Pharmaceutical therapies typically aim to manage infections and promote wound healing. These medications, like mupirocin, an antibiotic, and acidic fibroblast growth factor (aFGF) are advantageous for preventing and treating infections while also alleviating nipple pain [12]. Additional treatment options may involve antifungal agents to address candidiasis, a frequent condition linked to nipple fissures.

Non‐pharmacological therapies, on the other hand, are usually known as supportive care. Warm water compresses are commonly used to soothe the afflicted area [5, 16]. While expressed breast milk is often suggested, its efficacy varies significantly compared to alternative therapies [17, 18, 19]. For instance, a study comparing breast milk and coconut oil discovered that coconut oil had a better effect on reducing nipple fissures and pain severity compared to breast milk [18].

The other non‐pharmacological group includes traditional and herbal treatments. These treatments have higher compliance and easier acceptance by breastfeeding mothers due to their natural ingredients, but their efficacy has not been investigated thoroughly. Systematic reviews have identified various herbal medicines to be effective [2, 3, 5, 8, 16]. These include quince seed jelly, lanolin, olive oil, coconut oil, purslane, *aloe vera* , frankincense, pistacia atlantica (Saqez), curcumin, Ziziphus jujube, and other formulations [20, 21, 22, 23, 24, 25]. However, because there are insufficient well‐designed randomized controlled trials, physicians do not fully recommend these methods. Research indicates that only 0.6% of Chinese herbal medicine trials reported quality control measures for their interventions, while fewer than 10% clearly described randomization methods [26].

In comparison to other non‐pharmacological options, RepoGen cream showed superior efficacy in preventing and relieving nipple fissure symptoms. In our randomized controlled trial, RepoGen significantly reduced nipple pain, redness, wound, and bleeding by Day 3 (*p* < 0.001, *p* = 0.002, *p* < 0.001, and *p* = 0.015, respectively) and further improved redness and wound by Day 7 (*p* = 0.046 and *p* = 0.05, respectively). In this regard, quince seed jelly has yielded mixed results. Demirel and Kelek reported no significant benefit of quince seed in its traditional mucilage form over control, with efficacy apparent only in specialized nano‐hydrogel formulations combined with essential oils, limiting its practical application [18, 27, 28]. Coconut oil demonstrated reductions in pain and fissure severity compared to expressed breast milk, but this small, single‐blind trial limits its generalizability [20, 21, 22, 23, 24, 25, 29]. In terms of real‐world use, coconut oil is inexpensive and widely available yet lacks standardized dosing and purity, while quince seed preparations are regionally confined and often require complex processing. Other plant‐based therapies, such as *aloe vera* , purslane, and frankincense, have also been explored but suffer from marked heterogeneity in formulations, study designs, and outcome measures, reducing their reliability and accessibility in clinical practice.

### 4.2 The Rationale for Using Lanolin–Beeswax–Olive Oil Blend

Lanolin, a natural wax derived from sheep's wool, has traditionally been used topically to heal sore and cracked nipples [1, 2]. However, the evidence for its usefulness is still being debated. Shetty et al. [2] observed that lanolin significantly reduced nipple pain and damage. Dennis et al. [30] compared lanolin to an all‐purpose nipple ointment (APNO) and discovered no significant differences in pain scores, while women in the lanolin group were more satisfied with their newborn feeding approach. Jackson and Dennis found no significant difference in nipple pain scores between the lanolin and control groups 4 days after randomization; though both groups improved by Day 7. While more women in the lanolin group expressed satisfaction with the treatment, this did not translate into significant reductions in pain or breastfeeding results [4]. Based on these studies, it seemed that lanolin's protective effect materialized after a few days, and it might be useful to include additional ingredients in the mixture to increase its advantages even more.

Olive oil has high monounsaturated fatty acids, mainly oleic acid, and other bioactive substances, such as polyphenols and vitamins. These components potentially contribute to its emollient, antioxidant, and anti‐inflammatory qualities [31]. Tristanti et al. found a statistically significant reduction in nipple soreness in the olive oil group compared to the control group. However, the tiny sample size (15 responders) reduces the findings' generalizability [32]. Olive oil's emollient properties may soothe and moisturize cracked skin, lowering pain and facilitating quicker healing. Its antioxidant capabilities may also help protect the injured tissue from additional oxidative stress [31]. The anti‐inflammatory qualities may reduce inflammation associated with the fissure, resulting in pain alleviation, and tissue repair. While olive oil's advantages for many skin conditions are well documented, its specific efficacy in healing nipple fissures remains uncertain.

Honey has been shown to have wound healing capabilities, especially in the setting of nipple fissures; however, the solo effects of beeswax on nipple fissures have received less attention. Beeswax is a mixture of esters, fatty acids, and hydrocarbons with emollient, protecting, and antibacterial characteristics [33, 34]. These features point to the possible benefits of treating nipple fissures. Its protective nature may form a barrier against future irritation and infection [1]. Furthermore, its antibacterial qualities may assist in avoiding fissure infection, which is a frequent outcome [8, 35]. These antibacterial qualities of beeswax could provide an advantage over lanolin, which primarily serves as an emollient and protective barrier [8, 35]. Given the similarities in emollient and protective characteristics between beeswax and lanolin, it is possible that beeswax could provide similar benefits in curing nipple fissures. Both chemicals form a protective barrier to moisturize the skin and promote wound healing [1]. Overall, we report that applying a mixture of lanolin, beeswax, and olive oil significantly improves the results of nipple fissure symptoms, suggesting its effective application in wound healing.

### 4.3 Strengths and Limitations

Our study's strength is its comparative approach, which demonstrated the superiority of the RepoGen group across various factors, including nipple pain, redness, wound, bleeding, itching, breastfeeding frequency, and feeding sufficiency. We reported robust and reliable data as they were collected at two time points (i.e., Days 3 and 7). However, our study has some limitations, which should be considered when interpreting the findings. The study's limited sample size and lack of diversity could limit the findings' generalizability. The subjective nature of some outcome measures, such as pain levels, raises the possibility of reporting bias. Outcomes were assessed on Days 3 and 7 postpartum, and longer term effects may not have been captured. As many nipple fissures may develop or worsen after the first week, and complications such as recurrence, infection, or early breastfeeding discontinuation may occur later, our findings do not reach these longer term effects. Finally, a more thorough examination of side effects and long‐term safety issues is required.

## 5 Conclusions

The rationale for using RepoGen cream lies in the potential benefits of its ingredients. Lanolin, olive oil, and beeswax have emollient, protective, and antibacterial properties that may contribute to the healing of nipple fissures. RepoGen cream offers a rapid, safe, and effective means to alleviate early postpartum nipple pain and prevent wounds and bleeding, potentially reducing early breastfeeding cessation. Integration of RepoGen into standard postpartum care protocols could improve maternal comfort and breastfeeding success. Further multicenter trials comparing RepoGen directly with other moist‐healing agents, as well as cost‐effectiveness analyses, will solidify its role in clinical practice.

## Author Contributions

P.S. designed the study. S.Z.M. and M.N. analyzed the data and performed the statistical analyses. R.S., S.Z.M., F.M.M., A.M.S., and B.S.H. drafted the initial manuscript. S.B. assisted with the revisions based on the reviewers' comments. All authors reviewed the drafted manuscript for critical content. All authors approved the final version of the manuscript.

## Consent

The authors have nothing to report.

## Conflicts of Interest

The authors declare no conflicts of interest.

## Data Availability

The datasets analyzed during the current study are available upon request from the corresponding author.
